# Promises of Fibroblast Growth Factor Receptor–Directed Therapy in Tailored Cancer Treatment

**DOI:** 10.3390/jcm9082570

**Published:** 2020-08-08

**Authors:** Sharmila Fagoonee, Rinaldo Pellicano

**Affiliations:** 1 Institute of Biostructure and Bioimaging (CNR), Molecular Biotechnology Center, 10126 Turin, Italy; 2 Unit of Gastroenterology, Molinette Hospital, 10123 Turin, Italy; rinaldo_pellican@hotmail.com

Cancer, one of the deadliest and undefeatable diseases, involves the deregulated growth of cells with the conferment of a high potential to metastasize. It is a multifaceted disease with around 18 million cases diagnosed annually in the world [1]. In recent years, the discovery of oncogenic mechanisms regarding molecular drivers of cancer progression and response to treatment has led to the development of novel target therapies. In this regard, receptor tyrosine kinases (RTKs) have become an attractive therapeutic target. RTKs play a pivotal role in essential processes such as cell survival, migration, proliferation, and differentiation. Due to their importance in cellular homeostasis, the expression and activity of RTKs is finely tuned, and any aberrancy in their signalling leads to disease development. Chromosomal rearrangements, gene amplifications, gain of function, alternative splicing, and autocrine activation are among the most frequent causes of oncogenic RTK signalling [2,3]. Among the RTKs, the fibroblast growth factor–fibroblast growth factor receptor (FGF–FGFR) axis has attracted considerable attention as a druggable target, and several therapeutic strategies have been developed for its selective targeting in cancer [4].

FGFR was first recognized as an important entity in embryogenesis, and abnormalities affecting components of its pathway were associated with developmental defects and congenital malformations [5]. Since then, numerous studies have shown the fundamental role of the FGF–FGFR signalling pathway in various crucial biological processes including tissue regeneration and angiogenesis. Moreover, deregulation of FGFR signalling, through the above-cited oncogenic mechanisms, has been detected in cancer. For instance, aberrant FGF signalling, via upregulation of *FGFR 1, 2,* and *4* expression, has been implicated in cholangiocarcinoma (CCA) progression [6]. Several studies have also identified different *FGFR2* gene fusions in CCA cases, including *FGFR–BICCI, FGFR2–AHCYL1, FGFR2–TACC3,* and *FGFR2–KIAA* 1598 [7]. Importantly, in intrahepatic CCA, *FGFR2* fusions have been observed in 10% to 16% of patients by DNA sequencing of tumour samples [8]. These aberrations contribute to cancer cell proliferation, resistance to anticancer therapies, and neoangiogenesis; for example, the activation of downstream oncogenes (such as, enhanced *FRS2* and *STAT3* signalling) or the loss of microRNA regulation (such as loss of miR-99a, which modulates *FGFR3* expression) following *FGFR3–TCCC3* fusion are among the observed causes of such havoc [9].

Due to the high redundancy and pleiotropic effects of the FGF–FGFR signalling pathway, it has been challenging to design drugs targeting this axis. Therapeutic approaches to selectively block the FGF–FGFR system in human cancer rely on novel strategies and drugs, which are currently being employed in preclinical studies and clinical trials [10]. Tyrosine kinase inhibitors (TKIs), such as small molecules, antagonistic antibodies, or peptide inhibitors or FGF ligand traps, with high antitumour activity and specific toxicity profiles have been developed. Cytotoxic drugs can also be efficiently delivered into cancer cells through FGFRs. For instance, antibody–drug or ligand–drug conjugates or anti-FGFR aptamers can cause rapid downregulation of surface FGFRs and lead to cellular-trafficking-dependant lysosomal receptor degradation. Interestingly, in some cancers such as biliary tract cancers, FGFR genetic aberrations appear to be the most important predictor of sensitivity to small-molecule TKIs, such as NVP-BGJ398, a pan-FGFR inhibitor [11].

The development of resistance against applied therapeutics is a serious unresolved problem in cancer treatment. Tumour cells may evolve ways to bypass the FGFR–FGF signalling by overexpressing other RTK members (bypass signalling) or by inducing mutations in the inhibitor-binding pocket in the FGFR (gatekeeper mutations) [12]. In these cases, the use of combination therapies and novel FGFR therapies may help in overcoming cancer cell resistance. Moreover, FGFRs, which are not exposed on the cell membrane, may act independently of the tyrosine kinase activity. In fact, FGFRs have been described in several subcellular organelles, such as the endosome, nucleus, and mitochondria. Nuclear and mitochondrial FGFRs are capable of promoting tumourigenesis by modulating gene expression or cellular metabolism. Porębska et al. concisely reviewed how the trafficking of FGFRs can be targeted for selective cancer treatment [2]. As tyrosine kinase activity is not involved, the intracellular FGFRs cannot be targeted using classical anticancer drugs. New strategies aimed at inhibiting the cellular translocation of FGFRs are required. One strategy may involve the selective targeting of the processing proteases present at the plasma membrane [2].

Despite the effort in developing FGFR-pathway-targeting drugs, there are still barriers to their clinical application [13]. Epithelial-to-mesenchymal transition may emerge as a consequence of chronic exposure to FGFR inhibitors, hence resulting in resistance [14]. Additional layers of complication arise from alternative splicing of FGFRs by RNA-binding proteins causing alterations in ligand recognition. For instance, epithelial splicing regulatory factor-1 (*ESRP1*) was shown to promote the expression of the epithelial isoform of *FGFR2, FGFR-2-IIIb* (which binds specific ligands of the paracrine FGF family such as *FGF-7*), at the expense of the mesenchymal isoform FGFR-2-IIIc [3,15]. Both *FGFR2* and *ESRP1* are frequently amplified and demethylated in gastric cancer, hence leading to increased expression of *ESRP1* and the FGFR isoform IIIb [16]. A class switch from *FGFR-2-IIIb* to *FGFR-2-IIIc* has been observed during the progression of prostate and bladder cancer, with *FGFR-2-IIIc* being associated with a more malignant phenotype [17]. Interestingly, restoration of *FGFR-2-IIIb* in prostate cancer cells enhanced their sensitivity to radiation and chemotherapy, indicating that modulating alternative splicing may be a possible alternative to selectively target the FGFR–FGF pathway [16]. 

It is becoming increasingly clear that the consequence of FGFR aberrations is not uniform across cancer types. Molecular testing such as next-generation sequencing is employed to analyse tumours to detect actionable FGF/FGFR alterations, but biopsy location and tumour heterogeneity can limit this approach. There is, thus, an urgent need to develop biomarkers to choose the most appropriate treatment strategies and to frequently and non-invasively monitor response to targeted therapies [18]. Analysis of serum or urine circulating tumour DNA, faecal microRNAs, or extracellular vesicles released from tumour cells may overcome these limitations [19,20,21]. Endocrine FGFs may also be applied as serum biomarkers of cancer as recently reviewed in prostate cancer [15]. These data are encouraging and warrant further studies regarding the optimal timing and conditions for measuring these biomolecules.

As phase II clinical trials emerge, patient selection as well as methods for predicting response to therapy and for overcoming toxicity become essential. Future generations of FGFR inhibitors will hopefully overcome current barriers and expedite the availability of these new medications for blocking the progression of cancers that rely on the FGFR–FGF axis [13]. Understanding the FGFR status of tumours, as well as their natural history, molecular mechanisms, prognostic impact, and response to FGFR-targeting is crucial to offer precision-medicine-based therapy for their effective management. Thus, FGFR studies are an excellent example of how in-depth exploration at a molecular level could render the design of individualized therapy plans feasible [22].
